# Medical Quarrels

**Published:** 1842-03

**Authors:** 


*Medical Quarrels.*—On the old principle that "misery loves company," our readers may not be sorry to hear that all the medical squabbling is not confined to our city, nor even to our side of the Atlantic. Paris is at present the seat of a very pretty quarrel. It seems, M. Gerdy clinical Professor of the Faculty of Medicine, in a discourse lately pronounced by him at the opening of the session, has given mortal offence to some of the Parisians. Gerdy took for his theme, the life and character of Sanson, the lately deceased Professor of clinical surgery, and it cannot be denied that he did full and noble justice to his late colleague. The severe virtues of Sanson, his high and inflexible principle, his generous disinterestedness, his perfect honesty, are all well appreciated. This how ever need not have given offence and perhaps did not; but M. Gerdy went further, he exposed the ingratitude of Dupuytren, and his cold-hearted selfishness towards his pupil and his very devoted friend. Dupuytren it seems used Sanson in every way without scruple, availed himself of his literary talents, and yet never would forward his views, but rather used his influence to keep down Sanson, to keep him in the background. All this is brought out without ceremony by M. Gerdy—he also lets us into a piece of literary history, which is curious and amusing, viz: that Sanson *wrote part if not all of Dupuytren's Thesis* for the Concours at which he gained the Chair of Surgery. Dupuytren was, it appears, a slow and difficult writer, eternally revising, correcting and altering his manuscript—never satisfied—hence it happened that as the time for presenting the thesis approached, Dupuytren could not finish his, and actually wrote a letter to the Faculty, announcing his withdrawing from the Concours. Duprey, one of his friends, saw the letter and withdrew it. He afterwards persuaded Dupuytren to go forward, and then Sanson came forward in the fullness of his devotion to his master, finished the thesis, and thus opened to Dupuytren the career in which he gained so much fame and so plentiful a fortune. It is to this man who so signally *saved him* in his hour of need, that Dupuytren is said by M. Gerdy to have been ungrateful.

This accusation against their surgical idol, has united the Parisian Journals in a most furious tirade against Gerdy. He is denounced without stint or measure, and even without decency—meanwhile the facts will all probably come out, and we fear that it will be manifest that the great Parisian Surgeon was as deficient in moral qualities, as he undoubtedly was eminent for intellectual powers. Meanwhile, whatever may happen to the master, the pupil is sure to occupy a most enviable position. All parties unite in doing honor to Sanson, and he, who lived in poverty and suffering—who starved while Dupuytren was rioting in wealth, is now honored, beloved, venerated—and that, not because he was learned, or wise, or bold, or daring, but because he was good, because he was honest, because during his surgical llfe, he was ever under the influence of high moral principle, because no temptation ever induced him to swerve from the strait and narrow, and as he found it, the thorny path of strict professional honor and honesty.

Such was Sanson; he is dead—but *his* memory lives in green and beautiful freshness. Dupuytren too is dead—*his* memory lives, but alas! his friends are already reduced to the sad necessity of attempting to drown by clamor the voice of Gerdy, who has dared to tell that truth which none of them dare deny. There is a lesson in all this which ought not to be lost upon us—few can hope to rival the splendid achievements of Dupuytren, but all may practice the virtues of Sanson.—*N. Y. Med. Gaz.*

